# TMEM Proteins in Cancer: A Review

**DOI:** 10.3389/fphar.2018.01345

**Published:** 2018-12-06

**Authors:** Kathleen Schmit, Carine Michiels

**Affiliations:** URBC-NARILIS, University of Namur, Namur, Belgium

**Keywords:** cancer, TMEM proteins, biomarkers, tumor suppressors, oncogenes, chemoresistance

## Abstract

A transmembrane protein (TMEM) is a type of protein that spans biological membranes. Many of them extend through the lipid bilayer of the plasma membrane but others are located to the membrane of organelles. The TMEM family gathers proteins of mostly unknown functions. Many studies showed that TMEM expression can be down- or up-regulated in tumor tissues compared to adjacent healthy tissues. Indeed, some TMEMs such as TMEM48 or TMEM97 are defined as potential prognostic biomarkers for lung cancer. Furthermore, experimental evidence suggests that TMEM proteins can be described as tumor suppressors or oncogenes. TMEMs, such as TMEM45A and TMEM205, have also been implicated in tumor progression and invasion but also in chemoresistance. Thus, a better characterization of these proteins could help to better understand their implication in cancer and to allow the development of improved therapy strategies in the future. This review gives an overview of the implication of TMEM proteins in cancer.

## Introduction

A TMEM is a type of protein that spans the entire width of the lipid bilayer and to which it is permanently anchored. Many TMEMs function as channels to permit the transport of specific substances across the biological membranes. But the biological functions of many of them remain unknown mainly due to difficulties in the extraction and purification of these proteins. There are two ways to classify the TMEMs. The first one is according to their structure. Indeed there are two basic types of TMEMs, alpha-helical proteins and the beta-barrel proteins (Vinothkumar and Henderson, 2010). The second classification is according to their topology, this classification refers to the position of the N- and C-terminal domains (von Heijne, 2006).

Among TMEMs is the TMEM family. The proteins of this family are predicted to be components of various cell membranes, such as mitochondrial, endoplasmic reticulum, lysosome, and Golgi membranes. TMEMs are present in many cell types and fulfill important physiological functions such as epidermal keratinization (TMEM45A) (Hayez et al., 2014), autophagy, smooth muscle contraction (TMEM16) (Thomas-Gatewood et al., 2011), protein glycosylation (TMEM165) (Foulquier et al., 2012) and development and differentiation of the liver (TMEM97) (Malhotra et al., 1999). Among them, some members play a primordial role in immune response (TMEM9B) (Dodeller et al., 2008). Indeed, TMEM9B is a key component of inflammatory signaling pathways through the enhancement of the production of pro-inflammatory cytokines induced by TNF, IL1β, and TLR ligands.

In many cancers, differential regulation of the expression of TMEMs has been observed, such as in lymphomas (TMEM176) (Cuajungco et al., 2012), colorectal cancer (TMEM25) (Hrasovec et al., 2013), hepatic cancer (TMEM7) (Zhou et al., 2007), and lung cancer (TMEM48) (Qiao et al., 2016). Some of them are used as prognostic biomarkers. For example, in renal cancers, many TMEMs with predicted ER localization have been shown to be potential classifiers of cancer grade (e.g., TMEM45A, TMEM116, TMEM207, TMEM213…) (Wrzesinski et al., 2015). A large number of TMEMs have also been implicated in cancer development and in drug resistance, suggesting that the TMEM family is a prominent group for cancer research. Furthermore, some of these proteins act as tumor suppressors (e.g., TMEM25, TMEM7) (Zhou et al., 2007; Doolan et al., 2009) while others act as pro-oncogenes (e.g., TMEM158, TMEM14A…) (Cheng et al., 2015; Zhang et al., 2016). This review aims to describe the implication of the TMEM proteins in cancer.

## Part 1: TMEMs as Tumor Suppressors

Some TMEMs have been described in the literature to act as tumor suppressors. A downregulation of their expression is generally observed in tumor tissue compared to adjacent healthy tissue. It is for example the case for TMEM25. This protein is a member of the immunoglobulin super-family and is involved in immune response, growth factor signaling and cell adhesion (Katoh and Katoh, 2004). The expression of this protein has been studied in fresh tumor samples collected during surgical colectomy from patients who had been diagnosed with primary colorectal adenocarcinoma. TMEM25 mRNA expression was significantly decreased in 68% of tumor tissues in comparison to corresponding normal tissues. This downregulation has been correlated with the hypermethylation of a specific CpG site in the 5′ UTR region of TMEM25 gene in a high proportion of tumor samples (Hrasovec et al., 2013). Another study revealed that TMEM25 expression in the tumor tissues was lower than the one in normal healthy tissues in 50% of tumor samples in human breast tumor biopsies. The expression of TMEM25 was correlated with a better overall survival and associated with a longer survival time for patients who received adjuvant chemotherapy. Furthermore, in triple-negative breast tumors, TMEM25 was generally not expressed (Doolan et al., 2009). All together these findings suggest that TMEM25 may be used as a tumor biomarker of favorable prognosis.

Another example is TMEM7. This protein of 232 amino acids has a single transmembrane domain and is expressed in the liver. The gene coding for TMEM7 is localized in the short arm of chromosome 3, which is commonly deleted in cancer cells (Huebner, 2001). Chromosomal regions that are deleted in cancer are generally the loci of tumor suppressor genes, suggesting that TMEM7 is a candidate suppressor gene. This protein has been studied in 18 hepatocellular carcinoma cell lines but also in primary tumors obtained from surgical resection of hepatocellular carcinoma from 17 patients. Each tumor sample was matched with its corresponding healthy liver tissue. In the absence of homozygous deletion, TMEM7 is down regulated in 33% of the cell lines and 85% of the tumor samples compared to healthy tissue. Tumor suppressor genes located at chromosomal regions deleted in some cancer cells are found to be silenced by promoter methylation in other cell lines. In two lines of the latter that displayed TMEM7 downregulation, 5-aza-2′-deoxycytidine, a DNA methylation inhibitor and trichostatin A, a HDAC inhibitor, increased TMEM7 expression suggesting that aberrant methylation and histone deacetylation are responsible for the transcriptional silencing of this gene. The study of this protein also showed that INF-α induced TMEM7 mRNA expression and the restoration of its expression by overexpression or by induction with IFN-α decreased the proliferation and the invasion of hepatocellular carcinoma cell lines (SNU398 and PLC/PRF/5 or HLF and MHCC97 respectively). These data have also been validated *in vivo*. Indeed, ectopic expression of TMEM7 in two TMEM7 deficient hepatocarcinoma cell lines decreased tumor growth in nude mice (Zhou et al., 2007). All these data highlight the tumor suppressor role of TMEM7 in hepatocellular carcinoma.

Two recent studies also showed that TMEM176A could act as tumor suppressor. The first one was performed in esophageal squamous cell carcinoma. Wang et al. analyzed the methylation profile of TMEM176A promoter in 13 cell lines (BIC1, TE1, TE3, TE13, KYSE140, KYSE180, KYSE410, KYSE450, KYSE520, Segl, KYSE150, YES2, and COLO680N) and 267 primary esophageal squamous cell carcinoma. The results showed the loss of TMEM176 expression in 12 cell lines (TE1, TE3, TE13, KYSE140, KYSE180, KYSE410, KYSE450, KYSE520, Segl, KYSE150, YES2, and COLO680N) in association with a complete methylation of its promoter. It also revealed that 66% of primary tumors presented TMEM176A promoter methylation. This methylation and TMEM176A decreased expression were correlated with poor overall survival. The restoration in two cell lines, KYSE410 and KYSE150, of TMEM176A expression with 5′-aza-2′-deoxycytidine treatment and the downregulation of TMEM176A in BIC1 cells showed that TMEM176A inhibited cell invasion and migration and induced apoptosis. Furthermore, TMEM176A inhibited cell growth both *in vitro* and *in vivo* with a decrease in tumor volume when TMEM176A was re-expressed (Wang et al., 2017). A very similar study has been performed in colorectal cancer. It revealed that 50% of the primary tumors presented methylation of TMEM176 promoter. The results also showed a normal expression of TMEM176A in LS180 and SW620 cell lines, a decreased expression in HT29 and SW480 cell lines and a total loss of expression in LOVO, HCT116, RKO, and DLD1 cell lines respectively associated with no methylation, partial methylation and total methylation of TMEM176A promoter. In colorectal cancer as well as in esophageal squamous cell carcinoma, TMEM176A overexpression inhibited cell migration and invasion, induced apoptosis and inhibited cell growth both *in vitro* and *in vivo* (Gao et al., 2017). These two studies together presented TMEM176A as tumor suppressor of esophageal squamous cell carcinoma and colorectal cancer.

The last protein described in this part is TMEM97. This protein, also named MAC30, is a member of the insulin-like growth factor binding proteins (Murphy et al., 1993). TMEM97 mRNA is expressed in the fetal liver but not in adult liver suggesting a role in development and differentiation of the liver (Malhotra et al., 1999). In 2001 and 2002, two studies showed that the expression of TMEM97 can be induced by other genes like BRCA1 but also be downregulated by others like p53 suggesting that the expression of this gene can be deregulated in cancers (Kannan et al., 2001; Atalay et al., 2002). Indeed, the expression of TMEM97 is increased in several types of cancer as described later in this review, except in pancreatic and renal cancers that both display a low expression level of TMEM97 protein and mRNA. In 2004, 30 pancreatic cancer tissues obtained from patients after tumor resection and 19 non-cancerous pancreatic tissues obtained through an organ donor program have been used to analyze the expression level of TMEM97 in pancreatic cancer both at the mRNA level by RT-qPCR and at the protein level by histochemistry. 50% of pancreatic cancer biopsies displayed a lower TMEM97 mRNA expression compared to normal pancreatic tissue, 20% displayed no change and 30% presented higher TMEM97 mRNA levels. These results highlighted a high variability regarding TMEM97 expression levels in pancreatic cancer. A high variation in mRNA level expression was also observed in different pancreatic cancer cell lines (Aspc-1, BxPc-3, Capan-1, Colo-357, T3M4, Mia-PaCa-2 and Panc-1 cells). The protein expression and localization of TMEM97 were also analyzed: TMEM97 protein was strongly expression in the cytoplasm of islet cells and moderately in acinar cells. Cancer cells in pancreatic cancer tissues displayed weak or no expression of this protein in more than 75% of cases. But at low levels in pancreatic cancer cells (Aspc-1, BxPc-3, Capan-1, Colo-357, T3M4, Mia-PaCa-2 and Panc-1 cells). Knowing that tubular complexes are considered as potential pre-neoplastic lesions, The observed reduction of TMEM97 expression in pancreatic cancer suggests that this gene might act as a tumor suppressor in this disease (Kayed et al., 2004). This hypothesis may also be true for prostate cancer since miR-152-3p downregulation and promoter methylation were found to be prevalent in primary prostate cancers. TMEM97, which is overexpressed in this type of cancer, is a target of miR-152-3p (Ramalho-Carvalho et al., 2018).

## Part 2: TMEMs as Oncogenes

Many TMEMs are up regulated in cancer. Some of them are implicated in tumor progression, invasion and in the formation of metastasis while others are associated with poor prognosis and can be used as prognostic biomarker. The studies behind these conclusions are summarized here under.

### TMEMs as Prognostic Biomarkers

TMEM48, also named NDC1 is localized to the nuclear pore complexes. This nucleoporin has six membrane-spanning segments and is crucial for nuclear pore complexes and nuclear envelope assembly (Stavru et al., 2006). The integrity of the nuclear envelope and a correct nucleocytoplasmic transport are important for many cellular processes such as genome stability, DNA replication, or DNA repair (D’Angelo and Hetzer, 2008). Nucleoporin deregulation has been implicated in several malignancies such as breast cancers (Agudo et al., 2004; Kau et al., 2004) in multiple tumors including melanoma, pancreatic, colon, gastric, prostate, esophageal, lung cancer, and lymphomas (Mahipal and Malafa, 2016). A study based on 60 patients with NSCLC showed that TMEM48 expression was significantly higher in cancer tissues compared to healthy tissues. This overexpression was associated with poor prognosis, lymph node metastasis, increased tumor size and short survival (Qiao et al., 2016). All together these results suggest that, since TMEM48 mRNA expression is increased in non-small lung carcinoma in association with advanced tumor stage, TMEM48 may be a potential prognostic factor for NSCLC.

TMEM45A is a TMEM of 275 amino acids, predicted to have five to seven transmembrane domains and localized in the trans Golgi apparatus. Very little is known about this protein except that TMEM45A is highly expressed in the skin and is associated with epiderm keratinization (Hayez et al., 2014). This protein is overexpressed in many cancers: breast cancer, liver cancer, renal cancer, glioma, head and neck cancer, ductal cancer, and ovarian cancer (Flamant et al., 2012; Lee et al., 2012; Guo et al., 2015; Sun et al., 2015; Wrzesinski et al., 2015; Manawapat-Klopfer et al., 2016). In the cases of breast cancer and cervical lesions, a higher expression level of TMEM45A has been correlated with a lower patient overall survival suggesting that TMEM45A is a potential biomarker for aggressiveness of breast cancer and cervical lesions (Flamant et al., 2012; Manawapat-Klopfer et al., 2016).

Despite the putative tumor suppressor role of TMEM97 in pancreatic and prostate cancers, this protein is overexpressed in different types of cancer and associated with tumor progression, recurrence and poor survival. It is the case in breast, gastric, colon, epithelial ovarian, oral squamous, and NSCLC. Indeed, the expression of TMEM97 has been analyzed in 20 cases of NSCLC compared to adjacent healthy tissue: 65% of patients showed a higher expression level of TMEM97 in tumor tissue compared to healthy tissue. Furthermore, the expression of this protein has been correlated with poor tumor differentiation and a shorter patient survival (Han et al., 2013). A similar study performed in human SQCLC showed TMEM97 overexpression in 26 of the 32 tumor samples in comparison to corresponding non-tumor tissues. TMEM97 overexpression was associated with poor tumor differentiation and shorter overall patient survival (Ding et al., 2016). Another study in breast cancer revealed that 59.7% of tumor samples displayed a higher expression level of TMEM97 compared to healthy tissue and that this overexpression correlated with larger tumor size and tumor recurrences. One study on ovarian cancer showed that high expression of TMEM97 was correlated with high histological grade and tumor recurrence (Xiao et al., 2013; Yang et al., 2013). All these studies demonstrated that TMEM97 expression could affect the prognosis of NSCLC, SQCLC, ovarian and breast cancer patients.

Another important TMEM protein is TMEM16A. TMEM16A, also known as anoctamin-1, is expressed in cerebral artery smooth muscle cells and is predicted to have eight transmembrane domains. This protein is a TMEM that functions as a calcium-activated Cl- channel (Thomas-Gatewood et al., 2011). TMEM16A has recently been shown to be upregulated in several cancers including HNSCC, esophageal, breast and gastric cancers. In HNSCC, the expression of TMEM16A has been studied by fluorescence *in situ* hybridization and immunohistochemistry on several primary tumors. The results demonstrated that TMEM16A was highly expressed in 4–19% of the samples and that higher TMEM16A expression strongly correlated with poor prognosis of HNSCC patients (Ruiz et al., 2012). In another study in HNSCC, TMEM16A has been shown to be overexpressed in 84% of tumor samples (Carles et al., 2006). In the context of gastric cancer, the expression of TMEM16A has been evidenced to be higher in tumor tissue than in adjacent non-tumor tissue. Furthermore, the expression of this protein has been correlated with the tumor stage and negatively correlated with patient survival in this cancer type (Liu et al., 2015). TMEM16A is thus proposed to be a negative prognostic factor.

Two other TMEMs have been described as prognosis biomarker. In glioma, TMEM140 expression has been analyzed in 47 of the 70 glioma samples by immunohistochemistry. The results showed a higher expression in tumor tissue than in the control brain tissue and a correlation with poor prognosis in this cancer (Li et al., 2015a,b). In lung cancer, TMEM45B expression has been analyzed in 110 tumor tissue samples and 35 non-tumor tissue samples. TMEM45B was shown to be upregulated in lung cancer and its expression was negatively correlated with overall survival (Hu et al., 2016).

### TMEMs Involved in Tumor Growth

Besides the evidence for a correlation between TMEM expression and patient survival, some of these proteins have been shown to be directly involved in tumor growth but the mechanisms by which they act are not always known.

#### With an Identified Pathway

The first protein described in this part is TMEM158, also called Ras-induced senescence 1 protein (RIS1). The gene coding for this protein is known to be upregulated during Ras-induced senescence in human diploid fibroblasts infected with rasV12-containing retrovirus (Barradas et al., 2002). TMEM158 is overexpressed in Wilms tumors (also known as nephroblastoma) with somatic mutations in catenin beta-1 gene suggesting a relationship between the Ras and Wnt signaling pathways (Zirn et al., 2006). TMEM158 is also overexpressed in ovarian cancer in 84% of the 25 tumor samples which were analyzed. The involvement of TMEM158 in tumor growth has been studied in two ovarian cancer cell lines, HO-8910 and A2780. This protein was evidenced to regulate cell proliferation, adhesion, and invasion. Furthermore, TMEM158 knockdown inhibited tumor growth of HO-8910 cell line in nude mice highlighting the role of this protein in tumorigenicity. TMEM158 silencing led to the deregulation of the expression of different genes, including a downregulation of ICAM1 and VCAM1 expression. These two proteins are involved in cell adhesion. TMEM158 silencing also impaired the TGF-β signaling pathway (Cheng et al., 2015). All these results showed that TMEM158 may work as an oncogene in ovarian cancer.

The implication of TMEM48 in NSCLC progression has been studied in two cell lines that overexpressed this protein, A549 and H1299. The results suggested a role of TMEM48 in cell proliferation, migration and invasion. Indeed, the silencing of this gene impaired cell proliferation, induced cell cycle arrest and decreased the migration and invasive ability of NSCLC cells. The downregulation of TMEM48 also induced cell apoptosis in association with a decrease or an increase in anti- or pro-apoptotic gene expression respectively. One of these two cell lines (A549) was also used to study the involvement of TMEM48 in tumorigenicity *in vivo* and the data revealed that TMEM48 is involved in tumor formation from A549 cells in nude mice. A marked decrease in tumor weight (50%) was evidenced when TMEM48 was silenced. All these evidences showed a role of TMEM48 in lung cancer progression (Qiao et al., 2016). A recent study demonstrated that TMEM48 suppression by miR-421 increased the expression of the apoptotic and tumor suppressor proteins caspase 3, PTEN and p53 in A549 cells (Akkafa et al., 2018). These results suggest that TMEM48 modulates the apoptotic pathway.

TMEM14A is a TMEM with three transmembrane domains, localized in mitochondria. This protein is deregulated in different types of cancer such as ovarian cancer, colon cancer and hepatocellular carcinoma (Hodo et al., 2010; Smith et al., 2010; Zhang et al., 2016). In the context of ovarian cancer, TMEM14A is involved in cell proliferation as shown by a cell cycle arrest when TMEM14A was invalidated in two ovarian cancer cell lines, A2780 and HO-8910. TMEM14A up regulation also increased the cell invasive ability of ovarian cancer cells highlighting a potential role of this protein to promote metastasis. Further investigations showed that TMEM14A knockdown may down-regulate the protein expression of PCNA, cyclins and MMPs. It may also downregulate TGF-β signaling (Zhang et al., 2016). These results could explain the decrease in cell proliferation and invasiveness in ovarian cancer cell lines when TMEM14A was invalidated.

TMEM97 is found deregulated in several types of cancer but this protein has been particularly involved in the tumor growth of two cancers: glioma and gastric cancer. Indeed, the silencing of TMEM97 expression in glioma U373 and U87 cells inhibited cell proliferation and cell cycle progression associated with a decrease in cyclin B1, E, CDK2 and CDK4 expression, but also in cell invasiveness. TMEM97 silencing also induced the deregulation of the expression of EMT markers like β-catenin, Twist and E-cadherin (Qiu et al., 2015). The downregulation of TMEM97 in gastric cancer BGC-823 and AGS cell lines inhibited the cell proliferation and mobility with a decrease in Akt phosphorylation, hence suggesting that Akt may mediate the TMEM97-induced inhibition of proliferation (Xu et al., 2014). The invalidation of TMEM97 also induced an inhibition of cell migration and invasion by reducing the expression of cyclin B1 and WAVE2. These data showed that TMEM97 plays an important role in tumor growth and aggressiveness in glioma and gastric cancer.

Another TMEM protein involved in tumor growth is TMEM16A. In human colorectal cancer cells, the mRNA and protein expression of TMEM16A has been reported in several cell lines like SW620, HCT116 and LS174T but not in HCT8 and SW480. TMEM16A knockdown in SW620 cell line inhibited cell proliferation, migration and invasion. These effects were mediated through a decrease in the expression of cyclin D1 and in the phosphorylation of MEK and ERK1/2. Furthermore, invalidation of TMEM16A expression led to a delay in cell cycle progression (Sui et al., 2014). TMEM16A expression is also regulated epigenetically. Indeed, inhibition of HDAC class I and II by siRNA or pharmacological agents decreased the expression of TMEM16A. HDAC3 seems to be the most important one in this regard. Hence, the inhibition of HDAC3 may exert suppressive effect on cancer cell viability via the downregulation of TMEM16A in prostate or breast cancer (Matsuba et al., 2014). TMEM16A has also been well-studied in gastric cancer. Knockdown in AGS and BGC-823 gastric cancer cell lines inhibited cell migration and invasion via a downregulation of E-cadherin expression (EMT marker) probably via a decrease in TGF-β secretion since the supplementation of exogenous TGF-β restored E-cadherin expression and cell migration and invasion (Liu et al., 2015). TMEM16A silencing was also shown to induce apoptosis in human prostate cancer PC3 cells by upregulating TGF-β signaling (Song et al., 2018). In pancreatic ductal adenocarcinoma, TMEM16A is overexpressed in several cancer cell lines (Mia PaCa-2, Panc-1, BxPC-3, and AsPC-1) in comparison to HPDE-derived cells. The invalidation of TMEM16A expression in these cell lines using siRNA showed an implication of this protein in cell migration but not in the proliferation illustrating that TMEM16A modulates the metastatic potential of pancreatic cancer cells. Contrary to colorectal cancer, the molecular mechanism underlying this effect is still unknown (Sauter et al., 2015).

The last TMEM described in this part is a very peculiar TMEM protein, TMEM88. This protein is a potential 2-transmembrane type protein that interacts with an important component of Wnt signaling pathway: DVL1 (Lee et al., 2010). According to the localization of its partner DVL1, TMEM88 may be localized in the cytoplasm or to the plasma membrane. This protein is overexpressed in cancer tissue compared to non-cancerous tissue in different types of cancer such as in lung, colon, gastric, breast cancer (Yu et al., 2015; Zhang et al., 2015) and can be involved in the tumor initiation and progression through Wnt signaling pathway (Ge et al., 2018). For the majority of these cancer types, immunohistochemistry analysis demonstrated a cytosolic localization. But in the context of NSCLC, two different subcellular localizations for TMEM88 have been reported, suggesting different roles in tumor development depending on its localization. Indeed, an *in vitro* analysis on nine lung cancer cell lines (A549, H1299, H460, H292, SPC-A-1, LTEP-A-2, LK2, PG-BE1, and PG-LH7) showed that the overexpression of membrane-associated TMEM88 led to the inhibition of the canonical Wnt pathway through the downregulation of the expression of effectors like cyclin D1, MMP-7, and c-Myc. The increase in membrane-associated TMEM88 expression also led to a decrease of proliferation, colony formation, migration and invasion and to a decrease in tumor growth *in vivo* highlighting the tumor suppressor role of TMEM88 when it is localized to the membrane of the cell. Furthermore, TMEM88 promoter methylation is associated with unfavorable prognosis in NSCLC (Ma et al., 2017). On the contrary, its cytosolic localization is correlated with a low level of differentiation of the tumor and poor prognosis of patients with NSCLC. Furthermore *in vitro* analysis demonstrated that the overexpression or downregulation of this protein respectively enhanced or suppressed NSCLC cell migration and invasion through a deregulation of the EMT signaling pathway. Indeed, the TMEM88-DVL complex increased p38 and GSK3β phosphorylation leading to a stabilization of the protein SNAIL and hence to a decreased occludin and zonula occludens-1 (ZO-1) expression. Moreover, *in vivo* analysis showed that the number of lung metastatic nodules increased in the mice transplanted with cell lines expressing cytosolic TMEM88 (Zhang et al., 2015). Very similar results have also been observed in triple-negative breast cancer (Yu et al., 2015). These data confirmed that, in NSCLC and breast cancer, the cytosolic localization of TMEM88 conferred an oncogenic role to the protein.

**FIGURE 1:**
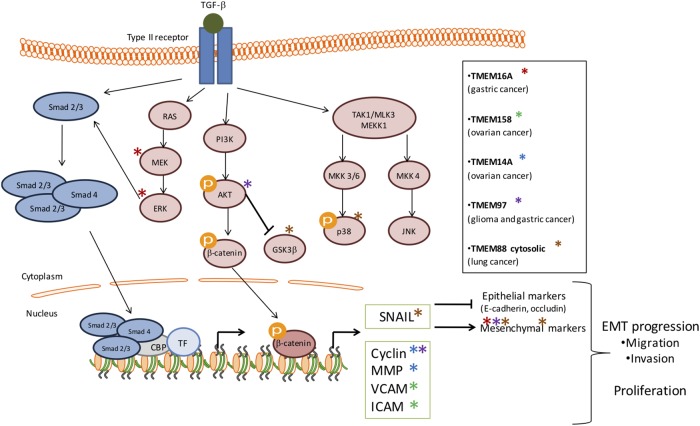
Schematic representation of the involvement of several TMEMs in tumor growth through the TGF-β signaling pathway. The activation of TGF-β signaling pathway has been implicated in many cellular processes and in tumor growth. This activation is induced by its ligand which then activates the phosphorylation of serine/threonine residues and triggers phosphorylation of the intracellular effectors, SMADs (blue). TGF-β receptors can also activate Smad-independent pathways (pink). In early stage TGF-β plays a tumor suppressor role whereas, in advanced stage, cancer cells benefit from TGF-β to initiate proliferation, invasion and metastasis dissemination. It seems that several TMEM proteins are involved in tumor growth through TGF-β activation in order to facilitate malignant progression and EMT progression. The stars represent the effectors deregulated by TMEMs.

Depending of cancer stage, TGF-β signaling can have different impact on tumor growth. Indeed, in early stage TGF-β plays a tumor suppressor role whereas in advanced stage, cancer cells benefit from TGF-β to initiate proliferation, invasion, and metastasis dissemination. It seems that several TMEM proteins are involved in tumor growth through TGF-β pathway modulation in order to facilitate malignant progression (Figure 1). Indeed, TMEM16A, TMEM158, TMEM14A, TMEM97, TMEM88 and probably TMEM45A interacts with several components of the TGF-β-induced signal transduction.

#### Through an Unknown Pathway

Other TMEMs have also an impact on tumor growth but the mechanisms by which they act are still unknown. Such an example is TMEM140 that is up regulated in cancer tissue compared to healthy tissue. TMEM140 has been involved in the regulation of the growth of glioma *in vitro* and *in vivo*. Indeed, when TMEM140 is silenced in two glioma cell lines *in vitro*, U87 and U373, the proliferation decreased with a higher proportion of cells in G1 phase and the cell viability decreased due to the activation of the apoptotic pathway. Furthermore, the knockdown of TMEM140 led to a decreased cell adhesion, migration and invasion. It has also been shown that the invalidation of this protein inhibited tumor growth *in vivo* with a decrease in the size and the weight of tumors in the invalidated group compared to the control group (Li et al., 2015a,b). These findings demonstrate that TMEM140 can be used as a prognosis biomarker but also as a therapeutic target.

Two other TMEM proteins have been involved in tumor progression, TMEM45A and TMEM45B, already described above. TMEM45A is implicated in cell proliferation, migration, and invasion of different cancers like glioma (U251 and U373 cells) and ovarian cancer (HO-8910 and A2780 cells) (Guo et al., 2015; Sun et al., 2015). In the context of ovarian cancer, TMEM45A protein expression has been positively correlated to TGF-β signaling pathway and this data could explain the impact of TMEM45A invalidation in this cancer (Guo et al., 2015). On the other hand, TMEM45B is up-regulated in human lung cancer and promotes tumorigenicity *in vivo*. Invalidation of TMEM45B in A549 and NCI-H1975 cells led to the inhibition of cell proliferation, migration, and invasion highlighting its role in tumor growth in lung cancer (Hu et al., 2016). In the case of pancreatic cancer, TMEM45B had also been involved in proliferation, invasion, and migration since its silencing in SW1990 and PANC-1 cell lines induced an inhibition of cell proliferation associated with cell cycle arrest. It also led to a decrease in cell mobility and invasiveness. Conversely, the overexpression of TMEM45B in CFPAC-1 cells promoted cell proliferation, invasion and migration (Zhao et al., 2016). TMEM45B is also upregulated in osteosarcoma cell lines. Its knockdown suppressed the prolifreation, migration, and invasion of U2OS cells *in vitro* as well as tumor growth in nude mice. These effects were associated with a decrease in the expression of β-catenin, cyclin D1 and c-Myc (Li et al., 2017). Similar results were obtained in gastric cancer cells, in which TMEM45B silencing was associated with a decrease in the abundance of p-STAT3 and p-JAK2 (Shen et al., 2018). These two proteins can be described as potential prognosis markers but also as regulators of tumor growth in several types of cancer.

## Part 3: TMEMs Involved in Chemoresistance

Although mutagenic alterations have long been associated with cancer development or drug resistance, epigenetic modifications and tumor microenvironment have also been linked to chemoresistance. Both epigenetic modifications and the tumor microenvironment can impact the expression or the localization of several TMEMs leading to a deregulation of treatment responses. The first example is hypoxia, one component of the tumor microenvironment. Indeed, in hypoxic condition (<1% of O_2_), hepatocellular carcinoma cells (HepG2) (Sermeus et al., 2008) and breast cancer cells (MDA-MB-231) (Flamant et al., 2010) were protected against cell death normally induced by chemotherapeutic drugs. In this condition, TMEM45A was shown to be upregulated and its silencing led to a decrease in this protective effect conferred by hypoxia against cell death induced by chemotherapeutic agents. These results suggest that, in hypoxic condition, TMEM45A is involved in the chemoresistance of breast and liver cancers. However, the mechanism underlying this protection is still unknown (Flamant et al., 2012).

The second example is related to epigenetic modifications, in particular DNA methylation. Indeed, in ovarian cancer, it has been shown *in vivo*, that the methylation profile of some promoters was different in xenografts resistant to cisplatin compared to control ones. This observation has been associated with a differential expression profile of the genes whose expression is regulated by these promoters. It is the case for TMEM88, which is a DNA methylation-regulated gene. The hypomethylation of TMEM88 promoter observed in ovarian cancer led to an increased expression of the protein and to platinum resistance. Indeed, knowing that TMEM88 was involved in Wnt signaling pathway, De Leon et al investigated the possible association of Wnt pathway and the observed phenotype. First of all, TMEM88 downregulation led to an increase in Wnt target gene expression such as β-catenin or Jun, validating the interaction between TMEM88 and Wnt pathway in ovarian cancer. Then, they studied the link between this interaction and the observed chemoresistance. TMEM88 overexpression in resistant cells inhibited the Wnt signaling pathway associated with a decrease in target gene expression while the activation of the Wnt pathway in resistant cells increased the chemosensitivity of the cells to cisplatin. Furthermore, the invalidation of TMEM88 in cisplatin resistant cells increased the sensitivity of the cells to the chemotherapeutic drug. This increase in chemosensitivity was associated to a decrease in cell proliferation allowing the escape of the cells from the genotoxic effects of cisplatin (de Leon et al., 2016).

Another TMEM involved in chemoresistance is TMEM205, also known as MBC3205. This protein of 21 kDa has four transmembrane domains and belongs to the group of secreted proteins (Clark et al., 2003). In 2011, a study revealed that TMEM205 is highly expressed in the pancreas, adrenal gland, liver, mammary gland and kidney (Shen et al., 2010). This study also showed that, in epidermoid carcinoma, this protein had the particularity to translocate in the presence of cisplatin. Indeed, TMEM205 is located at the cell surface but in the presence of the chemotherapeutic drug, the protein is translocated in an intracellular compartment at the periphery of the nucleus. Furthermore, its expression is increased in a cell line resistant to cisplatin and TMEM205 overexpression conferred resistance to cisplatin (Shen et al., 2010). Another study demonstrated that TMEM205 colocalized with RAB8, a marker of recycling endosomes. Interestingly, TMEM205 also colocalized with syntaxin 6 (STXR6), a regulator of protein trafficking, which is translocated at the same subcellular localization that TMEM205 in the presence of cisplatin. Then, the translocation of TMEM205 may allow the exocytosis of platinum containing vesicles, which thus results in the accumulation of the drug outside the cell (Shen and Gottesman, 2012).

In the tumor microenvironment, the immune system plays a crucial role that modulates tumor growth. Furthermore, cancer-associated inflammation also plays a role in chemoresistance (Chen et al., 2007). In this context, TMEM98, which has immune-related properties, mainly regarding the differentiation of T helper (Th) 1 cells, may be proposed as a novel chemoresistance-conferring gene (Fu et al., 2015). There are two RNA splicing forms of TMEM98 reported in the NCBI database, TMEM98-v1 and TMEM98-v2 respectively. Although there is a slight difference between them in the 5′ UTR sequence, their coding products are almost the same, which consists of 226 amino acids and a molecular weight of 24.6 kDa. In lung cancer, TMEM98 mRNA expression is higher in cancer tissues compared to healthy tissues. Furthermore, in two lung cancer cell lines, A549 and H460, the silencing of TMEM98 inhibited cell proliferation and suppressed the invasion and the migration of cancer cells meaning that this protein can have an impact in tumor growth (Mao et al., 2015). Knowing that tumor progression and chemoresistance can be accompanied with inflammation injuries and the link between TMEM98 and inflammation, this protein is a very interesting target for further investigations on anti-cancer drug resistance. In the case of hepatocellular carcinoma, TMEM98 has been identified as a chemoresistance-associated gene. Indeed, its expression is increased in two chemoresistant cell lines, MHCC97L/CisR and MHCC97L/DoxR resistant to cisplatin and doxorubicin respectively. Furthermore, the level of the upregulation increased with the degree of chemoresistance. This study also showed that TMEM98 mRNA expression was higher in tumor tissue of patients who received a transarterial chemoembolization treatment. Moreover, the patients who did not respond well to the treatment had higher TMEM98 expression level. These data demonstrated that this protein is involved in chemoresistance of hepatocellular carcinoma. In order to identify the mechanism of TMEM88 in chemoresistance, further investigation had been performed. In the absence of TMEM88 in resistant cell lines, a repression of activation of AKT in association with a repression of its downstream targets had been observed. Furthermore, the silencing of TMEM88 restored p53 phosphorylation and activation under cisplatin or doxorubicin treatment. These data showed that the chemoresistance induced by TMEM88 is associated with AKT activation and the repression of p53 activation (Ng et al., 2014).

The platinum-based chemotherapy is used for the treatment of several cancers such as lung cancer. In this model, the high expression level of TMEM97 has been correlated with the resistance of cancer to platinum-based treatment but also with poor patient survival (Chen et al., 2016; Ding et al., 2017). Indeed, Chen et al. (2016), showed that only 4% of patients with elevated expression of TMEM97 showed responses to therapy while 65% of patients with low expression of TMEM97 responded to the treatment. This study proposed TMEM97 as a biomarker of prognosis but also of the responses of NSCLC patients to chemotherapies.

Two other TMEMs could have an impact in chemoresistance via the immune system, TMEM176A and TMEM176B. These two proteins can physically interact one with the other and are both localized in the plasma membrane and vesicular intracellular compartments (Cuajungco et al., 2012). The expression of these two proteins is increased in lymphoma, which may allow the cancer cells to evade the immune system or negatively impact their detection by immune system (Cuajungco et al., 2012).

Knowing that many chemotherapeutic drugs induced cancer cell death, several TMEMs could also have an impact in chemoresistance by exerting an anti-apoptotic function. TMEM48 is such an example for lung cancer (Qiao et al., 2016), TMEM14A for ovarian cancer (Zhang et al., 2016) and TMEM45B for lung and pancreatic cancers (Hu et al., 2016; Zhao et al., 2016).

**FIGURE 2:**
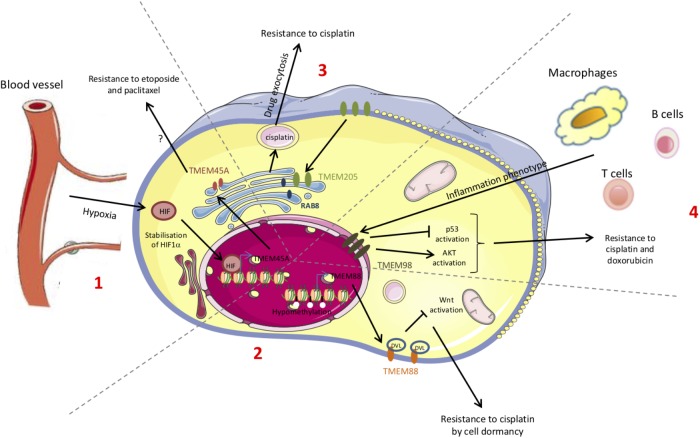
Schematic representation of the chemoresistance mechanisms conferred by TMEM proteins. The chemoresistance can be due to an adaptation of the cancer cells themselves (mutations, DNA methylation, proteins, translocation…) but can also be provided from the interactions with the microenvironment. Some of these chemoresistance mechanisms involve TMEM proteins. (1) Hypoxia leads to HIF-1α stabilization and to the expression of several target genes such as TMEM45A. (2) Methylation or acetylation of promoters leads to the transcriptional regulation of genes such as TMEM88. (3) The increase in TMEM205 expression and its translocation modify its partners. (4) The immune system induces the expression of several genes such as TMEM98.

The resistance to chemotherapy is not only due to the adaptation of cancer cells themselves but can involve tumor microenvironment. Furthermore, the mechanisms underlying the resistance to treatment can differ according to the cancer type and to the chemotherapeutic drug. The studies reported in this review showed that some TMEM proteins are involved in resistance to treatment and so can be used as new therapeutic targets (Figure 2). Finally, since TGF-β-induced quiescence renders cancer cells resistant to some anticancer agents (Brown et al., 2017; Tamai et al., 2017) and since many TMEM proteins interfer with TGF-β-induced intracellular signaling, TGF-β pathway is probably one of the key mechanisms through which TMEM proteins exert their effects.

## Conclusion

Despite the different role and localization of TMEM proteins, many of them are implicated in cancer (Table 1). Some of them can be correlated with stages and patient survival and so be used as biomarkers and/or classifiers. Others have a role in carcinogenesis and tumor progression, but for most of them, the mechanism involved is still unknown. A better characterization of these proteins could help to better understand their implication in cancer. A few of them are even involved in chemoresistance and could be used as new therapeutic targets to enhance the efficiency of chemotherapies.

**Table 1:** List of TMEM proteins involved in cancer.

TMEM	Localization	Function	Cancer			Models		Involvement in cancer	Reference
						
			Patient	*In vitro*	*In vivo*	*In vitro*	*In vivo*		
TMEM25	Unknown	Immune response	Colorectal adenocarcinoma	/	/	/	/	Tumor suppressor	Katoh and Katoh, 2004; Doolan et al., 2009; Hrasovec et al., 2013
TMEM7	Unknown	Interaction with olfactory receptors	Primary hepatocarcinoma	Hepatocarcinoma	Hepatocarcinoma	SNU398, PLC/PRF/5, HLF, MHCC97	SNU398, PLC/PRF/5	Tumor suppressor	Zhou et al., 2007
TMEM176A	Golgi apparatus (*cis*)	Unknown	Esophageal squamous cell carcinoma and colorectal cancer	Esophageal squamous cell carcinoma and colorectal cancer	Esophageal squamous cell carcinoma and colorectal cancer	BIC1, TE1, TE3, TE13, KYSE140, KYSE180, KYSE410, KYSE450, KYSE520, Segl, KYSE150, YES2, COLO680N and LS180, SW620, HT29, SW480, LOVO, HCT116, RKO and DLD1	KYSE410	Tumor suppressor	Gao et al., 2017; Wang et al., 2017
TMEM97	Unknown	Cholesterol level, growth and differentiation of the liver	Pancreatic cancer	Pancreatic cancer	/	Aspc-1, BxPc-3, Capan-1, Colo-357, T3M4, Mia-Paca-1, Panc-1	/	Tumor suppressor	Murphy et al., 1993; Malhotra et al., 1999; Kannan et al., 2001; Atalay et al., 2002; Kayed et al., 2004
			Ovarian, breast, lung cancer	Glioma and gastric cancer	/	U373, U87 and BGC-823, AGS	/	Oncogene/chemoresistance	Chen et al., 2007; Han et al., 2013; Xiao et al., 2013; Yang et al., 2013; Xu et al., 2014; Qiu et al., 2015; Ding et al., 2016, 2017
TMEM48	Nuclear pore complexes	Assembly and insertion of nuclear pore complexes to the nuclear membrane	Lung carcinoma	Lung carcinoma	Lung carcinoma	A549, H1299	A549	Oncogene	Agudo et al., 2004; Kau et al., 2004; Stavru et al., 2006; D’Angelo and Hetzer, 2008; Qiao et al., 2016
TMEM45A	Golgi apparatus (trans)	Association with epidermal keratinization	Breast, liver, renal, head and neck, ductal, ovarian cancers and glioma	Glioma, hepatocellular carcinoma, ovarian, breast, cancers	/	U251, U373, HO-8910, A2780, HepG2, MDA-MB231	/	Oncogene/chemoresistance	Sermeus et al., 2008; Flamant et al., 2010, 2012; Lee et al., 2012; Hayez et al., 2014; Guo et al., 2015; Sun et al., 2015; Wrzesinski et al., 2015; Manawapat-Klopfer et al., 2016
TMEM16A	Plasma membrane	Calcium activated chloride channels	Head and neck, esophageal, breast, prostate, gastric, colorectal cancer	Head and neck, gastric, colorectal cancers	/	Cal-27, Cal-33, BHY, SW620, HCT116, LS174T, AGS, BGC-823	/	Oncogene	Carles et al., 2006; Thomas-Gatewood et al., 2011; Ruiz et al., 2012; Sui et al., 2014; Liu et al., 2015; Sauter et al., 2015
TMEM140	Unknown	Unknown	Glioma	Glioma	/	/	/	Oncogene	Liu et al., 2015; Li et al., 2015a
TMEM158	Unknown	Hypothetical function in a neuronal survival pathway	Ovarian cancer	Ovarian cancer	Ovarian cancer	HO-8910, A2780	HO-8910	Oncogene	Barradas et al., 2002; Zirn et al., 2006; Cheng et al., 2015
TMEM14A	Mitochondria	Inhibition of apoptosis	Hepatocellular carcinoma, ovarian and colon cancers	Ovarian cancer	/	A2780, HO-8910	/	Oncogene	Hodo et al., 2010; Smith et al., 2010; Zhang et al., 2016
TMEM88	Plasma membrane	Inhibition of Wnt/beta-catenin signaling pathway (membrane associated) and heart development	Lung, breast, colon cancers and hepatocellular, gastric carcinoma	Lung cancer	Lung cancer	A549, H1299, H460, H292, SPC-A-1, LTEP-A-2, LK2, PG-BE1 and PG-LH7	LK2	Tumor suppressor if membrane associated/chemoresistance	Lee et al., 2010; Yu et al., 2015; Zhang et al., 2015; de Leon et al., 2016; Ge et al., 2018
	Cytosolic						A549, H1299	Oncogene if cytosolic	
TMEM45B	Unknown	Unknown	Lung cancer	Lung, pancreatic cancers	Lung cancer	A549, NCI-H1975, SW1990, PANC-1	A549	Oncogene	Hu et al., 2016; Zhao et al., 2016
	Unknown	Unknown	Osteosarcoma	Osteosarcoma	Osteosarcoma	U2OS	U2OS	Oncogene	Li et al., 2017
	Unknown	Unknown	Gastric cancer	Gastric cancer	/	BGC-823,MGC-803, SGC-7901, HGC-27	/	Oncogene	Shen et al., 2018
TMEM205	Plasma membrane or perinuclear	Hypothetical role in secretion or vesicular trafficking	/	Epidermoid carcinoma	/	KB-3-1, KB-CP.3, KB-C.5, Balb/3T3	/	Oncogene/chemoresistance	Clark et al., 2003; Shen et al., 2010; Shen and Gottesman, 2012
TMEM98	Unknown	Unknown	Lung cancer and hepatocellular carcinoma	Lung cancer and hepatocellular carcinoma	Hepatocellular carcinoma	A549, H460, MHCC97L/CisR, MHCC97L/DoxR	MHCC97L/CisR, MHCC97L/DoxR	Oncogene/chemoresistance	Ng et al., 2014; Fu et al., 2015; Mao et al., 2015


*The table summarizes their localization, function and the type of cancer in which they are implicated, with the corresponding references.*

## Author Contributions

KS wrote the review and designed the figures and the table. CM supervised the whole work, contributed to writing, and critically revised the paper.

## Conflict of Interest Statement

The authors declare that the research was conducted in the absence of any commercial or financial relationships that could be construed as a potential conflict of interest.

## Abbreviations

BRCA1: breast cancer type 1 susceptibility protein
CDK: cyclin-dependent kinase
DVL1: disheveled 1
EMT: epithelial–mesenchymal transition
ERK: extracellular signal-regulated kinase
GSKβ: glycogen synthase kinase 3β
HDAC: histone deacetylase
HNSCC: head and neck squamous cell carcinoma
HPDE: normal pancreatic ductal epithelium
ICAM: intercellular adhesion molecule 1
IFN: interferon
IL: interleukin
MAC30: meningioma-associated protein
MEK: mitogen-activated protein kinase kinase
MMP: matrix metalloproteinase
mRNA: messenger ribonucleic acid
NDC1: transmembrane Nucleoporin
NSCLC: non-small cell lung cancer
PCNA: proliferating cell nuclear antigen
RAB8: Ras-related protein
SQCLC: squamous cell lung carcinoma
STXR6: syntaxin 6
TLR: toll-like receptor
TGF-β: transforming growth factor-β
TNF: tumor necrosis factor
TMEM: transmembrane protein
UTR: untranslated region
VCAM: vascular cell adhesion molecule 1
ZO-1: zona occludens 1
